# The Hydrophobin HYTLO1 Secreted by the Biocontrol Fungus *Trichoderma longibrachiatum* Triggers a NAADP-Mediated Calcium Signalling Pathway in *Lotus japonicus*

**DOI:** 10.3390/ijms19092596

**Published:** 2018-09-01

**Authors:** Roberto Moscatiello, Simone Sello, Michelina Ruocco, Ani Barbulova, Enrico Cortese, Sebastiano Nigris, Barbara Baldan, Maurizio Chiurazzi, Paola Mariani, Matteo Lorito, Lorella Navazio

**Affiliations:** 1 Department of Biology, University of Padova, Via U. Bassi 58/B, 35131 Padova, Italy; roberto.moscatiello@unipd.it (R.M); sello.simone@gmail.com (S.S.); enrico.cortese.1@phd.unipd.it (E.C.); barbara.baldan@unipd.it (B.B.); mariani@bio.unipd.it (P.M.); 2 Institute for Sustainable Plant Protection, CNR, Via Università 133, 80055 Portici (NA), Italy; michelina.ruocco@ipsp.cnr.it; 3 Institute of BioSciences and BioResourses, CNR, Via P. Castellino 111, 80131 Napoli, Italy; ani@arterrabio.it (A.B.); maurizio.chiurazzi@ibbr.cnr.it (M.C.); 4 Botanical Garden, University of Padova, Via Orto Botanico 15, 35123 Padova, Italy; sebastiano.nigris@unipd.it; 5 Department of Agricultural Sciences, University of Napoli “Federico II”, Via Università 100, 80055 Portici (NA), Italy; lorito@unina.it

**Keywords:** aequorin, biocontrol fungi, calcium signalling, *Lotus japonicus*, hydrophobins, HYTLO1, NAADP, *Trichoderma*

## Abstract

*Trichoderma* filamentous fungi are increasingly used as biocontrol agents and plant biostimulants. Growing evidence indicates that part of the beneficial effects is mediated by the activity of fungal metabolites on the plant host. We have investigated the mechanism of plant perception of HYTLO1, a hydrophobin abundantly secreted by *Trichoderma longibrachiatum*, which may play an important role in the early stages of the plant-fungus interaction. Aequorin-expressing *Lotus japonicus* suspension cell cultures responded to HYTLO1 with a rapid cytosolic Ca^2+^ increase that dissipated within 30 min, followed by the activation of the defence-related genes *MPK3*, *WRK33*, and *CP450*. The Ca^2+^-dependence of these gene expression was demonstrated by using the extracellular Ca^2+^ chelator EGTA and Ned-19, a potent inhibitor of the nicotinic acid adenine dinucleotide phosphate (NAADP) receptor in animal cells, which effectively blocked the HYTLO1-induced Ca^2+^ elevation. Immunocytochemical analyses showed the localization of the fungal hydrophobin at the plant cell surface, where it forms a protein film covering the plant cell wall. Our data demonstrate the Ca^2+^-mediated perception by plant cells of a key metabolite secreted by a biocontrol fungus, and provide the first evidence of the involvement of NAADP-gated Ca^2+^ release in a signalling pathway triggered by a biotic stimulus.

## 1. Introduction

*Trichoderma* is a widely spread genus of free-living filamentous fungi belonging to Ascomycota, increasingly used in agricultural applications as biocontrol agents and biofertilizers [1,2,3,4,5]. The effects exerted by *Trichoderma* at the plant level are both indirect, due to the mycoparasitic activity on a plethora of phytopathogens, and direct, due to the induction of plant defence responses and promotion of growth and development [6]. For its peculiar lifestyle traits, plant root colonization and favourable impact on plant physiology, *Trichoderma* has been defined as an opportunistic, avirulent plant symbiont [7]. Although the beneficial effects produced by *Trichoderma* on the plant have been extensively demonstrated, little is known about the molecular mechanisms underlying signal transduction during the early stages of the interactions of plants with these biocontrol fungi. The induction of pathogen resistance is accomplished through the secretion of a complex arsenal of fungal molecules encompassing cell wall-degrading enzymes, secondary metabolites with antibiotic activity, and other substances [8]. Culture filtrates of *Trichoderma atroviride*, grown alone or in co-culture with the phytopathogen *Botrytis cinerea*, have been shown to induce in soybean cell cultures transient elevation in cytosolic free Ca^2+^ concentrations and defence responses, such as reactive oxygen accumulation and programmed cell death [9]. Nevertheless, the molecular mechanisms causing the observed effects on the plant host has been determined only for a few of the many secreted metabolites found in *Trichoderma* [10]. 

Calcium is a universal signalling element involved in a plethora of transduction pathways in all eukaryotes [11,12,13], as well as in prokaryotes [14]. In plants, Ca^2+^ has been demonstrated to mediate a wide array of signalling cascades in response to both abiotic and biotic stimuli [15,16,17]. Changes in intracellular free Ca^2+^ levels are a common early event during many plant-microbe interactions, of both symbiotic and pathogenic nature [18]. In particular, the role of Ca^2+^ signalling in the establishment of plant defence responses has been known for many years [19]. 

In this work we have investigated the mechanism of plant perception and transduction of HYTLO1, a hydrophobin abundantly secreted in the culture medium by *Trichoderma longibrachiatum* strain MK1 [20]. Hydrophobins are a family of small amphypathic proteins found exclusively in filamentous fungi, which are known to mediate the interactions between the fungus and its environment. In addition to having a general role during formation of aereal hyphae, sporulation, and production of fruit bodies, hydrophobins are thought to be involved in plant-fungus interactions, both pathogenic and symbiotic, by mediating the adhesion of fungal hyphae to the root surface [21]. Information about the role of hydrophobins in biocontrol fungi in plant root colonization, induction of plant defences, antifungal activity against phytopathogens, as well as responses to abiotic stresses is rapidly emerging [20,22,23]. A *Trichoderma* mutant, defective for the *TasHyd1* gene encoding a hydrophobin, although unaffected in its mycoparasitic activity, was found to be unable to colonize the plant root apparatus [24]. 

By using the model legume *Lotus japonicus* [25,26] stably expressing the genetically encoded Ca^2+^ indicator aequorin, we demonstrated that HYTLO1 triggers in plant cells a signal transduction pathway leading to the activation of defence genes in a Ca^2+^-dependent manner. Experiments performed by using the chemical probe Ned-19 [27] showed that the Ca^2+^ signalling pathway activated by HYTLO1 is mediated by nicotinic acid adenine dinucleotide phosphate (NAADP), a metabolite of nicotinamide adenine dinucleotide phosphate (NADP) which has been demonstrated to act as a potent Ca^2+^ mobilizing messenger in a wide variety of eukaryotes (see [28] for a review), including plants [29], as well as green and brown macroalgae [30,31]. The obtained data provide the first evidence for the involvement of this pyridine nucleotide-based Ca^2+^ agonist in a physiological event in higher plants and offer new insights into the mechanism of action of fungal hydrophobins.

## 2. Results

### 2.1. Generation of Transgenic *L. japonicus* Plants Stably Expressing the Bioluminescent Ca^2+^ Reporter Aequorin

To investigate the potential participation of calcium in the plant perception of HYTLO1, we transformed *Lotus japonicus* with a cDNA construct encoding the bioluminescent Ca^2+^ reporter aequorin. *L. japonicus* is a well-characterized model legume, widely used as an experimental system to analyse different types of plant-microbe interactions along the symbiosis-pathogenesis spectrum [32]. The aequorin coding sequence was cloned between the Cauliflower Mosaic Virus 35S promoter (CaMV 35S) and the *Agrobacterium tumefaciens* nopaline synthase terminator (tNOS) sequences in the pCAMBIA vector [33] to obtain the T-DNA construct pAB1 (Figure S1A). PAB1 was then used for *Agrobacterium tumefaciens*-mediated transformation-regeneration procedure of *L. japonicus* [34,35] wild-type and *Ljsym4-2* mutant, where the latter is impaired in the symbioses with both arbuscular mycorrhizal (AM) fungi and rhizobia [36]. Transgenic plants were selected on hygromycin-containing medium and allowed to self-pollinate. Several independent lines of the first generation of transformants (T1), showing a hygromycin resistance segregation of 3:1 were tested by semi-quantitative RT-PCR to confirm aequorin expression and analyse the amount of transcript in the different transgenic lines. Homozygous plants of the second generation (T2), exhibiting the highest aequorin expression (Figure S1B), were selected to set up in vitro cell cultures. Suspension-cultured cells are particularly useful to understand some complex plant physiological processes, such as Ca^2+^-mediated signal transduction events [37,38].

### 2.2. The Hydrophobin HYTLO1 Triggers a Transient Cytosolic Ca^2+^ Elevation in Aequorin-Expressing *Lotus japonicus* Cells

**Figure 1:**
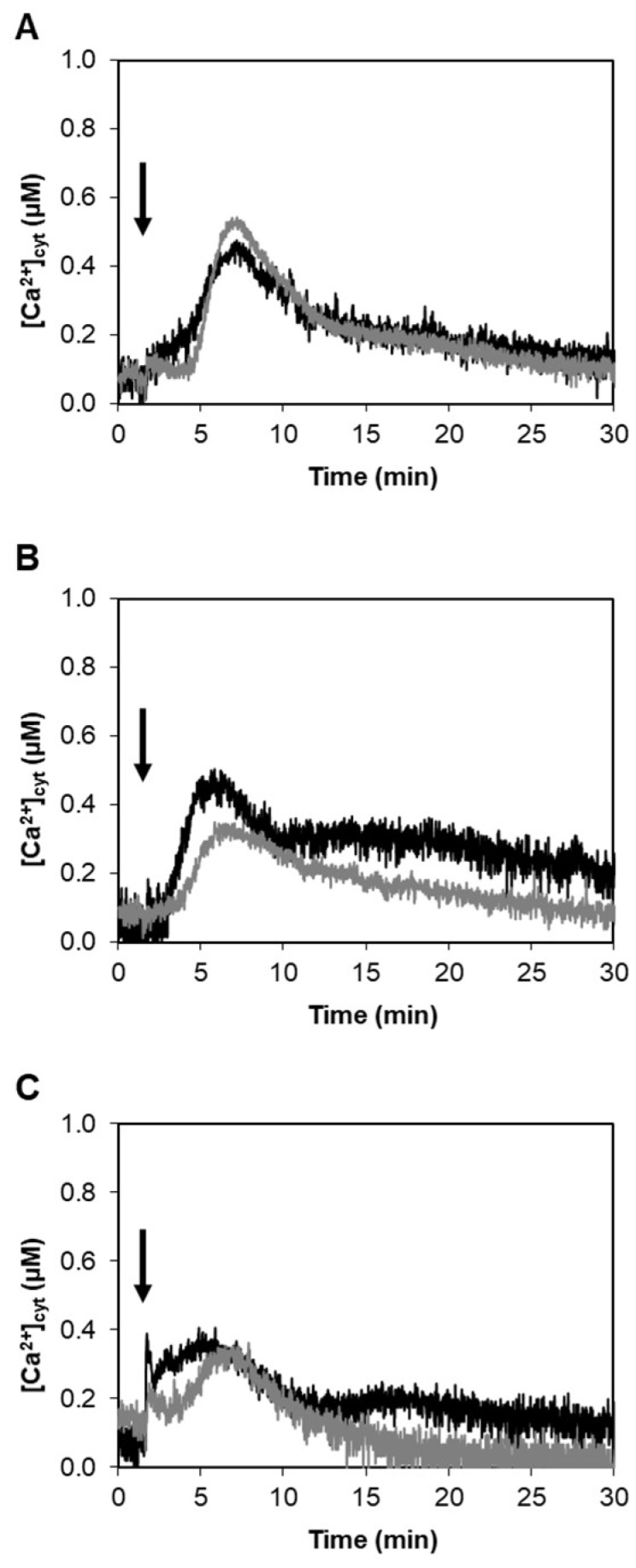
Monitoring of cytosolic Ca^2+^ concentration ([Ca^2+^]_cyt_) in aequorin-expressing *L. japonicus* in response to different biotic stimuli. Suspension-cultured cells of *L. japonicus* wild-type (black trace) and *Ljsym4-2* mutant (grey trace), stably transformed with a cDNA construct encoding cytosolic aequorin, were treated with: (**A**) the hydrophobin HYTLO1 (0.6 µM) purified from the biocontrol fungus *T. longibrachiatum*; (**B**) *Myc factors* from the AM fungus *G. margarita*; (**C**) *Nod factors* from the nitrogen-fixing symbiotic bacterium *M. loti*. Arrows indicate the time (100 s) of stimulation. These and the following traces are representative of at least 6 independent experiments that gave similar results.

HYTLO1, the major hydrophobin secreted by the strain MK1 of *Trichoderma longibrachiatum*, has been previously isolated from the culture medium of the biocontrol fungus, purified to homogeneity, and cloned [20]. To check if the perception and signal transduction of this protein involves calcium as intracellular messenger, aequorin-expressing *L. japonicus* cell suspension cultures derived from the transgenic plants were challenged with HYTLO1. The hydrophobin (0.6 µM) was found to trigger a transient cytosolic Ca^2+^ increase that peaked (0.48 ± 0.09 µM; n = 10) about 6 min after the injection and slowly dissipated within 30 min. The kinetics of the cytosolic Ca^2+^ change ([Ca^2+^]_cyt_) was very similar in *L. japonicus* wild-type cells and *Ljsym4-2* mutant cells, stably expressing cytosolic aequorin (Figure 1A). The *Ljsym4-2* mutant is defective in the *CASTOR* gene that encodes a cation channel essential for nuclear and perinuclear Ca^2+^ spiking in legume root endosymbiosis [39,40,41]. 

As a comparison, the cytosolic Ca^2+^ transients evoked by the symbiotic signalling molecules Myc factors and Nod factors, produced by the arbuscular mycorrhizal fungus *Gigaspora margarita* and the specific rhizobial symbiont *Mesorhizobium loti* are respectively shown in Figure 1B,C. In these latter two cases the Ca^2+^ traces exhibited by the *Ljsym4-2* mutant differ from the wild-type for the lack of the second, flattened dome-shaped [Ca^2+^]_cyt_ elevation (after about 15 min), which is consistent with the sum of the perinuclear Ca^2+^ oscillations that were visualized in Ca^2+^ imaging experiments in *L. japonicus* wild-type, but not *CASTOR* mutant [39].

### 2.3. HYTLO1 Activates the Expression of Defence Genes in *L. japonicus* Cells, but Does Not Induce Cell Death

**Figure 2:**
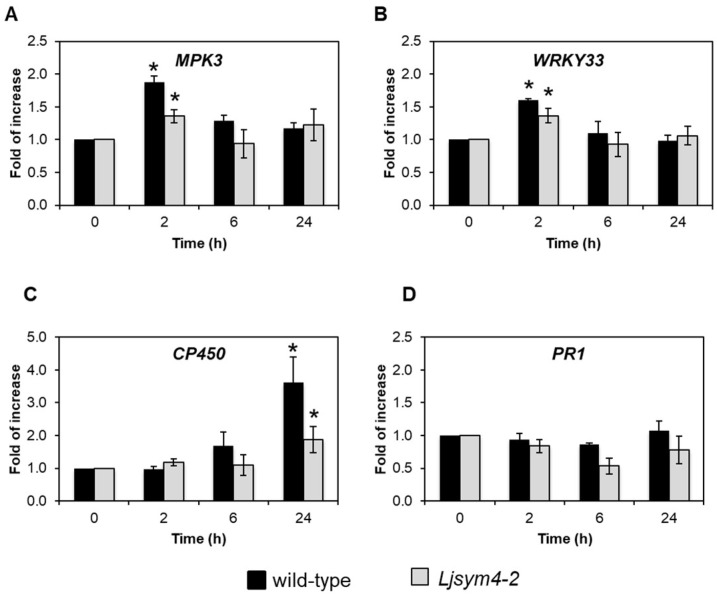
Analysis of HYTLO1-induced gene expression in *L. japonicus* cells. Semi-quantitative RT-PCR analysis of *L. japonicus MPK3* (**A**), *WRK33* (**B**), *CP450* (**C**), and *PR1* (**D**) in aequorin-expressing *L. japonicus* cell cultures of the wild-type line (black bars) and *Ljsym4-2* mutant line (grey bars) in control conditions and after treatment with HYTLO1 (0.6 µM) for different time intervals. Data are the means ± SE of three independent experiments. * indicates statistically significant difference at *P* < 0.05.

We next evaluated the effect of the treatment of *L. japonicus* cells with HYTLO1 on the expression of some genes commonly involved in plant defence against phytopathogens, namely *MPK3*, encoding the mitogen-activated protein kinase 3, *WRKY33*, encoding the transcription factor WRK33, *CP450*, encoding the cytochrome P450, and *PR1*, encoding the pathogenesis-related protein 1. Exponentially growing *L. japonicus* cells of the wild-type line and *Ljsym4-2* mutant line were treated with 0.6 µM HYTLO1 for 2 h, 6 h, and 24 h. Semi-quantitative RT-PCR analyses conducted with the wild-type cells showed a variable range of responses to the HYTLO1 application. *MPK3* and *WRKY33* were significantly up-regulated (1.88 ± 0.09 and 1.61 ± 0.02, respectively) after 2 h of treatment, with a subsequent decay to basal expression levels in the following 24 h (Figure 2A,B). The *CP450* gene, although remaining at constitutive level after 2 h of treatment with HYTLO1, gradually increased its expression after 6 h (1.67 ± 0.43) up to more than a three-fold induction level compared to the control (3.61 ± 0.78) after 24 h (Figure 2C). On the other hand, the expression of *PR1* was not significantly modified by the treatment with 0.6 µM HYTLO1 for any of the considered time intervals (Figure 2D). Concerning the *Ljsym4-2* mutant line, the gene expression analysis showed a trend similar to that observed in the wild-type line, but with lower values (Figure 2A–D). 

The results concerning the statistically significant differences between HYTLO1-treated samples and controls were validated by quantitative RT-PCR (Figure S2). Taken together, these data indicate that HYTLO1 can be considered as a mild elicitor of plant defence responses. 

**Figure 3:**
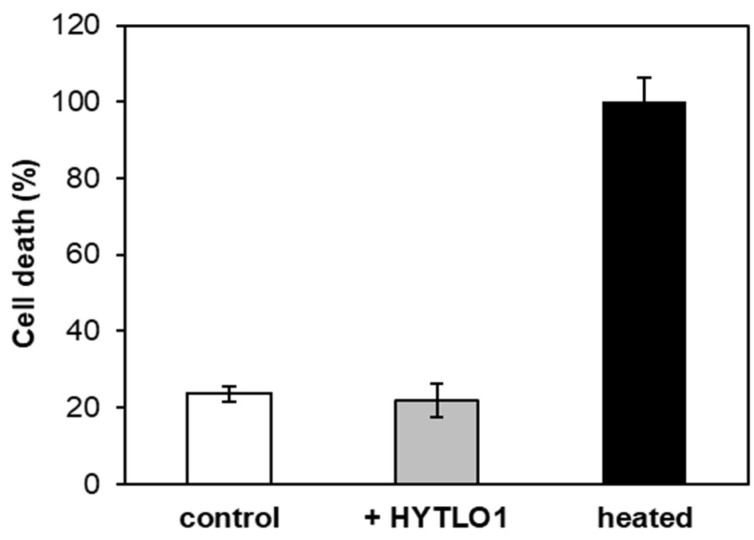
Effect of HYTLO1 on *L. japonicus* cell viability. Cell suspension cultures were treated with 0.6 µM HYTLO1 (grey bar) for 24 h. Cells incubated with the same percentage of ethanol (white bar) were used as control. 100% cell death corresponds to cells incubated for 20 min at 100 °C (black bar). Cell viability was determined by the Evans blue method. Data are the means ± SE of three independent experiments.

To evaluate the potential cytotoxicity of the fungal hydrophobin, we tested the viability of *L. japonicus* cells by using the Evans blue method [42]. This colorimetric assay revealed that the percentage of dead cells after treatment with HYTLO1 at 0.6 µM for up to 24 h did not significantly differ from that of control cells (Figure 3).

### 2.4. Origin of the HYTLO1-Elicited Cytosolic Ca^2+^ Fluxes in *L. japonicus*

**Figure 4:**
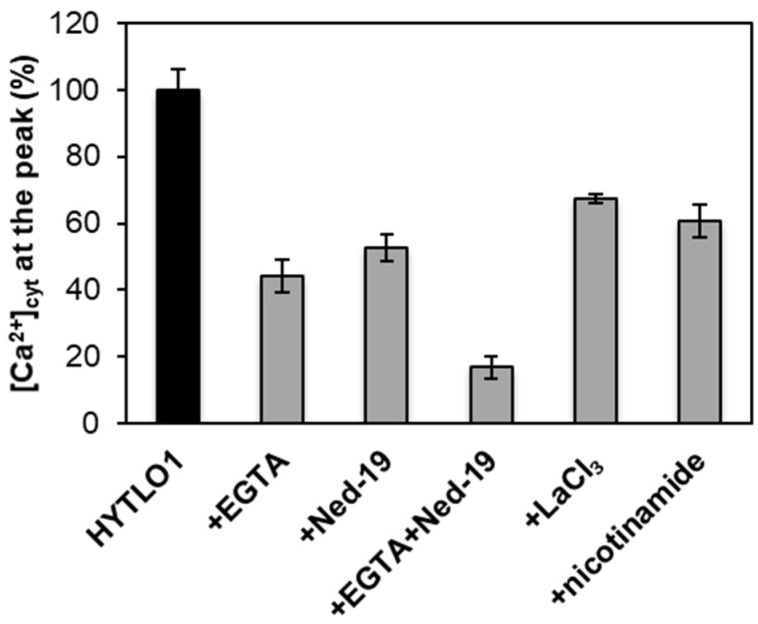
Pharmacological approach for the analysis of cytosolic Ca^2+^ fluxes induced by HYTLO1 in *L. japonicus* cells. Cell samples were incubated with: EGTA (600 μM), Ned-19 (100 μM) (singly or in combination with EGTA), LaCl_3_ (3 mM), nicotinamide (100 μM). After 10 min (EGTA, LaCl_3_, nicotinamide) or 30 min (Ned-19), cells were challenged with HYTLO1 (0.6 μM). For assays performed with EGTA, cells were previously washed extensively and resuspended in Ca^2+^-free medium. Data are the means ± SE of five independent experiments.

To assess whether the transient elevation in [Ca^2+^]_cyt_ induced by HYTLO1 in *L. japonicus* plays a key role in the signalling pathway leading to the activation of defence genes, the effect of the abolition of the Ca^2+^ change on downstream responses was analysed. Experiments based on the use of Ca^2+^ chelators, inhibitors of Ca^2+^ channels and of enzymes involved in the generation of Ca^2+^ mobilizing agents were carried out in the attempt to effectively block the HYTLO1-induced [Ca^2+^]_cyt_ transient. When cells, 10 minutes before HYTLO1 administration, were transferred to a culture medium depleted of CaCl_2_ and containing 600 μM EGTA, about 56% reduction of the [Ca^2+^]_cyt_ peak was observed (Figure 4). Pre-treatment with 100 μM Ned-19, a competitive antagonist of the intracellular Ca^2+^-mobilizing agent NAADP [27], caused inhibition of ~47% of the Ca^2+^ transient (Figure 4). These data indicate that in *L. japonicus* cells HYTLO1 mobilizes Ca^2+^ from both the extracellular space and from an intracellular compartment sensitive to NAADP. Moreover, the two stores (external and internal) seem to be involved almost at the same extent as sources of the ion for the transduction of this signal. As expected, the [Ca^2+^]_cyt_ elevation evoked by HYTLO1 was very efficiently blocked (83.2 ± 3.5%) by pre-treating *L. japonicus* cells with EGTA (600 μM) in combination with Ned-19 (100 μM) in Ca^2+^-free medium (Figure 4). LaCl_3_ (3 mM), a widely used inhibitor of Ca^2+^-permeable channels located at the plasma membrane [43], caused a reduction of only ~33% of the HYTLO1-Ca^2+^ transient (Figure 4), suggesting that additional Ca^2+^ channels are involved in the Ca^2+^ influx activated by HYTLO1 from the extracellular medium. Nicotinamide (100 μM), an inhibitor of ADP-ribosyl cyclase, involved in the production of both cyclic ADP-ribose (cADPR) and NAADP [44,45], reduced by ~40% the [Ca^2+^]_cyt_ in response to HYTLO1 (Figure 4), confirming the participation of NAADP-gated Ca^2+^ channels in the generation of HYTLO1-induced Ca^2+^ fluxes.

### 2.5. HYTLO1-Induced Activation of Defence Gene Expression is Ca^2+^-Dependent

**Figure 5:**
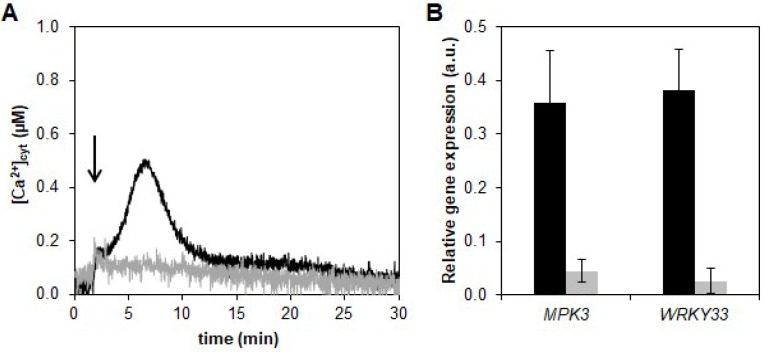
Effect of pretreatment with EGTA and Ned-19 on HYTLO1-induced [Ca^2+^]_cyt_ transient and gene expression in *L. japonicus* cells. (**A**) Monitoring of [Ca^2+^]_cyt_ in *L. japonicus* after treatment (arrow) with 0.6 µM HYTLO1 in regular cell culture medium (black trace) or after pretreatment with 600 µM EGTA and 100 µM Ned-19 in Ca^2+^-free medium (grey trace). (**B**) RT-PCR analysis of the expression of *MPK3* and *WRKY33* after treatment with HYTLO1 (0.6 µM) for 2 h in regular medium (black bar) or in Ca^2+^ free medium supplemented with 600 µM EGTA and 100 µM Ned-19 (grey bar).

In view of the efficient inhibition of the HYTLO1-induced Ca^2+^ transient obtained by the pre-treatment of *L. japonicus* cells with 600 μM EGTA and 100 μM Ned-19 in Ca^2+^-free medium (Figure 5A), the same experimental condition was used to examine the effect of the abolition of the HYTLO1-induced Ca^2+^ signalling pathway at the level of gene expression. The change of expression of the defence genes *MPK3* and *WRKY33*, observed after 2 h treatment of wild-type cells with 0.6 μM HYTLO1 (Figure 2A,B), was reduced by more than 85% in the presence of EGTA + Ned-19 (Figure 5B). This result demonstrates that the activation of *MPK3* and *WRKY33* gene expression in response to HYTLO1 is Ca^2+^-dependent. 

Even the expression of the *CP450* gene after 24 h HYTLO1 treatment was found to be significantly inhibited (~95%) by pre-treatment with EGTA + Ned-19 (Figure S3A). However, this result must be interpreted with caution; in fact, despite treatment with HYTLO1 not causing, by itself, any significant change in cell viability compared with the control (Figure 3), the pre-treatment with EGTA + Ned-19 caused, after 24 h, a significant rise of the cell death (Figure S3B). These data indicate the uselessness of a gene expression analysis after such a prolonged incubation time of the suspension-cultured cells with the above Ca^2+^ chelator and Ca^2+^ channel inhibitor.

### 2.6. HYTLO1 Perception Occurs at the Plant Cell Surface

Negative staining with 1% uranile acetate of a preparation of HYTLO1, dissolved in 50% ethanol, showed that this protein, similarly to other type II hydrophobins [20], forms in aqueous solution spherical air mycelles by autoassembling in amphypatic monolayer (Figure S4). Immunofluorescence analysis of HYTLO1-treated *L. japonicus* cells carried out with affinity-purified polyclonal antibodies raised against the purified protein indicated that HYTLO1 interacts with the plant cell surface, with no evidence for protein internalization inside the cell within 24 h (Figure S5).

**Figure 6:**
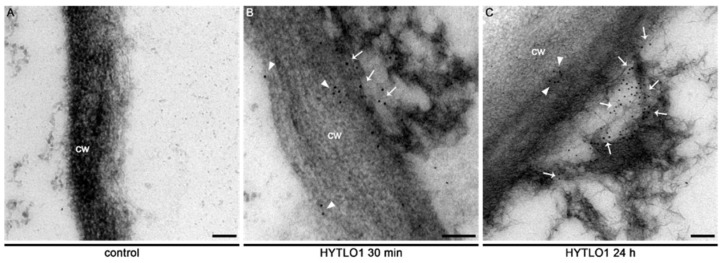
Immunocytochemical analysis of *L. japonicus* cells treated with HYTLO1. Cells were incubated: (**A**) in control conditions; (**B**) with HYTLO1 (0.6 µM) for 30 min; (**C**) with HYTLO1 (0.6 µM) for 24 h. Immunogold labelling was carried out by incubation with an affinity-purified anti-HYTLO1 antibody followed by a secondary antibody conjugated with 10 nm diameter-gold particles. Immunogold-labelled particles are visible at the external surface (arrows) and across the plant cell wall (arrowheads). cw, cell wall. Bars, 100 nm.

Immunogold labelling observations showed that HYTLO1 covers the plant cell wall external surface by forming a protein film (Figure 6), with some evidence for the permeation of the fungal hydrophobin across the plant cell wall (Figure 6 and Figure S6). 

**Figure 7:**
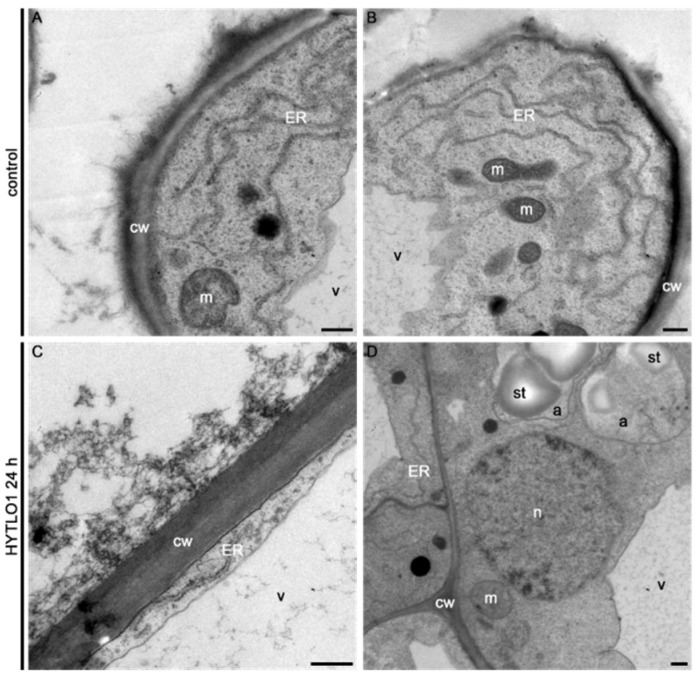
Electron microscopy observations of HYTLO1-treated *L. japonicus* cells. Cells were incubated: (**A**,**B**) in control conditions; (**C**,**D**) in the presence of HYTLO1 (0.6 µM) for 24 h. The ultrastructure of cells was found to be well-preserved even after 24 h treatment with the fungal hydrophobin. a, amyloplasts; cw, cell wall; ER, endoplasmic reticulum; m, mitochondria; n, nucleus; st, starch granules; v, vacuole. Bars, 500 nm.

In agreement with cell viability data (Figure 3), transmission electron microscopy (TEM) analyses demonstrated that the ultrastructural organization of *L. japonicus* cells was well preserved after 24 h treatment with HYTLO1 (Figure 7).

## 3. Discussion

Hydrophobins are small molecular weight proteins that play multiple roles in the cell biology of filamentous fungi [46]. In particular, during the early phases of plant-fungus interactions they may play an important function by mediating adhesion to the root surface [21]. Nevertheless, their potential role as elicitors of plant defence responses has been relatively little investigated so far. 

In this work we have demonstrated that HYTLO1, a hydrophobin abundantly secreted by the biocontrol fungus *Trichoderma longibrachiatum*, is perceived in a Ca^2+^-dependent manner by *L. japonicus* suspension-cultured cells by inducing a fast, transient [Ca^2+^]_cyt_ (Figure 1A) and the subsequent activation of genes commonly considered as hallmarks of plant defence responses (Figure 2A–C). Unlike complex metabolite mixtures isolated from *Trichoderma* culture filtrates, which have been previously shown to cause programmed cell death in soybean cell cultures [9], no reduction in cell viability or ultrastructural alterations were observed after treatment of *L. japonicus* cells with HYTLO1 (Figure 3 and Figure 7). On the other hand, our data suggest that the fungal hydrophobin acts as a mild proteinaceous elicitor, which may be able to pre-alert plant defence prior to a potential subsequent attack by a real pathogen—a mechanism commonly defined as “priming” [47]. 

Negative staining of preparations of pure HYTLO1 suggested the assembly of the protein in monolayers (Figure S4), that may help the fungus break the air-water interface during the early stages of plant-fungus interactions. Indeed, the peculiar physical features of these fungal proteins [48] have recently attracted a great deal of interest in view of their potential biotechnological applications as natural surfactants [49]. Unlike other hydrophobins, such as HYDPt-1 from the basidiomycete *Pisolithus tinctorius* that is exclusively located at the surface of fungal hyphae [50], HYTLO1 is secreted by *T. longibrachiatum* strain MK1 and interacts with the plant cell surface, as demonstrated by immunofluorescence (Figure S5) and immunogold labelling experiments (Figure 6 and Figure S6) with a specific antibody raised against the purified native protein.

The research work previously carried out by Lopez-Gomez et al. [51] in the same model legume *L. japonicus* highlighted the complex interplay of defence responses and symbiotic signalling pathways. Indeed, the notion that boundaries between pathogenesis and symbiosis are subtle and fluid is rapidly emerging [52]. Consistently, the *Ljsym4-2* mutant, which is characterized by an early block of the common symbiotic signalling pathway induced by both Myc factors and Nod factors, has shown altered gene expression profiles both in control conditions and after treatment with different biotic stimuli in several additional studies [53,54].

The new experimental system set up in this work, given by aequorin-expressing suspension-cultured cells of *L. japonicus*, derived from both wild-type and *Ljsym4-2* mutant, can be a useful tool to analyse Ca^2+^ signalling events not only during the beneficial interaction with an avirulent symbiont, such as the biocontrol fungus *Trichoderma* [7], but also in the establishment of symbioses with rhizobia and AM fungi. The *Ljsym4-2* mutant is defective in the *CASTOR* gene encoding for a K^+^-permeable channel that has been clearly demonstrated to be an essential modulator of the Ca^2+^ spiking phenomenon originating in the nuclear and perinuclear region of legume cortical cells in response to endosymbiotic microbes [39,41,55,56]. The molecular identity of the nuclear-localized Ca^2+^ channels responsible for symbiotic Ca^2+^ oscillations has recently been unravelled [40]. Indeed, when the two *L. japonicus* cell lines were challenged with the microbial symbiotic molecules Myc factors and Nod factors, the [Ca^2+^]_cyt_ traces observed in the wild-type and mutant cell lines were clearly different, i.e., the *Ljsym4-2* lacked the second flattened Ca^2+^ increase occurring after about 15 min (Figure 1B,C). This differential Ca^2+^ response is likely due to the lack of the asynchronous cytosolic Ca^2+^ oscillations, stemming from the controlled Ca^2+^ release/uptake by the nuclear envelope in the mutant cell population, when compared to the wild-type [39,57,58]. On the other hand, the trace and kinetics of the cytosolic Ca^2+^ change triggered by the hydrophobin was found to be very similar in wild-type and *Ljsym4-2* mutant cell lines (Figure 1A). This was generally true also for the changes of expression of genes involved in plant defence (Figure 2 and Figure S2). These results indicate possible differences in the signalling transduction pathways triggered by factors secreted by different symbiotic partners, which can be consistent with the versatility of plant receptor-like kinases (RLKs) involved in the response to microbial signals [59]. On the basis of our TEM analyses of an HYTLO1-derived web-like network at the plant cell surface, we may hypothesize that the observed intracellular Ca^2+^ elevation and change in defence gene expression can originate, at least in part, by a mechanical stimulation exerted by the fungal biofilm. Mechanical stimuli generate Ca^2+^ signals in plants, and the specific plant mechanosensing mechanisms are biological processes that have recently attracted the attention of different research groups [60,61]. Moreover, it has been demonstrated that plant perception of soft mechanical stress can activate defence responses [62]. However, TEM observations of HYTLO1-treated *L. japonicus* cultured cells favoured the possibility that the 7.2 kDa hydrophobin [20] may also permeate the apoplastic compartment (Figure 6 and Figure S6), possibly interacting with the plasma membrane. Indeed, *Trichoderma* secretes in vivo a battery of cell wall degrading enzymes [63], which may further facilitate the access of secreted molecules, and then HYTLO1, to specific binding sites at the plasma membrane level. Further work will be required to identify potential receptors for hydrophobins. 

The pharmacological approach used in this work suggests that HYTLO1 triggers both a Ca^2+^ influx from the extracellular milieu, as well as a Ca^2+^ release from a NAADP-sensitive intracellular Ca^2+^ store. NAADP is a pyridine nucleotide derivative that in the last two decades has been increasingly demonstrated to act as a potent Ca^2+^ mobilizing agent in animal cells, in addition to the well-established intracellular messenger inositol 1,4,5-trisphosphate (InsP_3_) and cyclic ADP-ribose (cADPR) [28]. Although NAADP has been shown to trigger Ca^2+^ release also in higher plants [29], its involvement in plant physiological events had yet to emerge. By using Ned-19, a newly-developed chemical probe for NAADP [27], a NAADP-gated Ca^2+^ release was shown to occur in response to copper excess in the marine alga *Ulva compressa* [30], as well as in the brown alga *Ectocarpus siliculosus* [31]. These data suggest that the spectrum of photosynthetic organisms responsive to NAADP might be broader than previously envisaged. The results obtained in this work confirm that Ned-19 can be used as a valuable tool to investigate NAADP-mediated Ca^2+^ signalling and highlight the participation of NAADP in a plant response to a biotic stimulus (a fungal proteinaceous elicitor). Interestingly, whereas members of the two-pore channel (*TPC*) gene family have been demonstrated to encode NAADP receptors in acidic organelles (endo-lysosomes) of animal cells (see [64,65] for review), the identity of NAADP receptors in plant cells still remain elusive. *TPC1*, the only member of the *TPC* gene family in *A. thaliana*, has been shown to encode the slow-vacuolar (SV) channel, a Ca^2+^-permeable channel located at the vacuolar membrane [66] and regulated by voltage and Ca^2+^ (see [67,68,69] for review). Interestingly, NAADP was found to be completely ineffective in the activation of TPC1, as well as of other tonoplast cation channels [70]. Indeed, early biochemical studies, consisting in ^45^Ca^2+^ release assays on sucrose-fractionated membrane vesicles, indicated an endoplasmic reticulum (ER)-localization for NAADP-gated Ca^2+^ release [29]. Interestingly, the recently reported crystal structure of *A. thaliana* TPC1 highlighted the presence of a Ned-19 binding site [71], suggesting the potential ability of this pharmacophore to allosterically block the channel activation. However, Ned-19 does not interact with the functionally-relevant voltage sensor [72]. Future in depth-studies will be necessary to further investigate the intracellular localization and additional physiological roles of plant NAADP receptors.

## 4. Materials and Methods

### 4.1. Chemicals

The hydrophobin HYTLO1 was isolated and purified to homogeneity from in vitro cultures of the biocontrol strain MK1 of *Trichoderma longibrachiatum*, as described by Ruocco et al. [20]. The pure protein was dissolved in 50% ethanol. Crude Myc factors were obtained by collecting the exudates of germinated spores of the arbuscular mycorrhizal fungus *Gigaspora margarita* (kindly provided by Paola Bonfante, Torino, Italy), lyophilizing and resuspending them in cell culture medium prior to cell treatment, as previously described [57]. Crude Nod factors extracted from *Mesorhizobium loti* were kindly provided by Makoto Hayashi (Yokohama, Japan). All chemicals used in the pharmacological analysis of Ca^2+^ fluxes (EGTA, LaCl_3_, nicotinamide) were obtained from Sigma-Aldrich (St. Louis, MO, USA), except *trans*-Ned-19, that was purchased from ENZO Life Sciences (Farmingdale, New York, USA).

### 4.2. Plant Material

*Lotus japonicus* (ecotype Gifu) wild-type and *sym4-2* mutant seeds, defective in *CASTOR*, were kindly provided by J. Stougaard (Aarhus, Denmark) and M. Parniske (Munich, Germany), respectively.

### 4.3. Molecular Cloning and *L. japonicus* Transformation Procedures

To obtain the aequorin-expressing T-DNA construct, the aequorin cDNA was excised from the cytAEQ plasmid [73] by *Sma*I digestion. The 600 bp *Sma*I fragment was subcloned into the pSE380 plasmid (Thermo Fisher Scientific, Waltham, MA, USA) in order to gain additional restriction sites for the next step of subcloning. A *Bgl*II-*Sal*I fragment was then ligated between a CaMV-35S promoter sequence and a tNOS terminator into pCAMBIA1300 [33] *Bgl*II-*Sal*I double digested to obtain the pAB1 T-DNA construct (Figure S1A). 

*Agrobacterium tumefaciens*-mediated *L. japonicus* transformation was performed as previously described [34,35]. Primary transformed plants of both wild-type and *Ljsym4-2* symbiotic mutant lines were selected on 5 µg/mL hygromicin B-containing medium and allowed to self-pollinate. Successful transformation and expression of the construct was confirmed by RT-PCR analysis of aequorin gene expression. Selected T2 homozygous plants (Figure S1B) were used to set up in vitro cell cultures.

### 4.4. Establishment of Aequorin-Expressing *L. japonicus* Cell Cultures

Aequorin-expressing cell cultures were set up by in vitro dedifferentiation of hypocotyls from transgenic *L. japonicus* seedlings. Briefly, hypocotyl explants from 8-day-old axenically grown seedlings were transferred on agarized (0.8%, *w*/*v*) Callus Induction Medium (CIM, 3.2 g/L Gamborg B5 basal medium, 0.5 g/L MES, 2% (*w*/*v*) sucrose, 0.5 µg/mL 2,4-dichlorophenoxyacetic acid (2,4-D), 0.05 µg/mL kinetin), supplemented with 5 µg/mL hygromycin B. After two subculturing steps, well-developed calli were transferred in Gamborg B5 liquid medium, pH 5.5, containing 2% (*w*/*v*) sucrose, 2 μg/mL 2,4-D, 5 μg/mL hygromycin B. Suspension-cultured cells were maintained at 24 °C on a rotary shaker at 80 rpm under 16 h light /8 h dark photoperiod. They were subcultured weekly by inoculating 1 mL packed cell volume into 20 mL fresh cell culture medium with, as described by Moscatiello et al. [37].

### 4.5. Cell Treatments

Exponentially-growing cells (five days) were treated with purified HYTLO1 (0.6 μM). The dose applied to cells was chosen on the basis of previous work concerning in vivo bioassays of physiological effects of HYTLO1 on plants [20]. In some experiments cells were treated with crude preparations of Myc factors or Nod factors. The final dose applied to cells corresponded to 10-fold concentrated AM fungal exudates and 1 µM Nod factors. In this latter case, suspension-cultured cells were grown in low nitrogen medium (containing 5 mM KNO_3_) for two days prior to the onset of the Ca^2+^ measurement assays.

### 4.6. Aequorin-Based Ca^2+^ Measurements

Aequorin was reconstituted by overnight incubation of mid-exponential phase transgenic *L. japonicus* cell cultures with 5 µM coelenterazine (Prolume, Pinetop, AZ, USA) in darkness. Cells were then extensively washed with fresh culture medium and allowed to recover from the touch response for at least 15 min. Aequorin-based Ca^2+^ measurements were performed as recently described [38], by using 100 µL of reconstituted cell suspension culture (containing about 5 mg cells, fresh weight). For pharmacological studies, cells were pre-incubated with the appropriate inhibitor for 10 min (LaCl_3_) or 30 min (Ned-19, nicotinamide). For experiments carried out in the absence of extracellular Ca^2+^, cells were extensively washed with Ca^2+^-free culture medium and then resuspended in the same medium containing 600 µM EGTA. In the case of molecules dissolved in ethanol (HYTLO1) or DMSO (Ned-19), control cells were treated with the same percentage (maximum 0.5%, *v*/*v*) of the organic solvent.

### 4.7. Immunofluorescence, Immunocytochemisty, and TEM Analyses

Immunofluorescence experiments were performed on *L. japonicus* suspension cultured cells as described by Zonin et al. [74]. Labelling was carried out by using affinity-purified polyclonal antibodies raised against purified native HYTLO1 (ProteoGenix, Schiltigheim, France), diluted 1:100, followed by Alexa Fluor 594 goat anti-rabbit IgG (Thermo Fisher Scientific). Working concentrations and specificity of the primary antibody were assessed in Western blot experiments against purified HYTLO1 and crude protein extracts from *L. japonicus* cells. 

TEM analyses were performed as described by Tagu et al. [50]. Immunogold labelling (dilution 1:100) was performed as recently described [75]. Samples were observed with a Tecnai 12-BT transmission electron microscope (FEI, Eindhoven, The Netherlands) operating at 120 kV equipped with a Tietz camera.

For negative staining, purified HYTLO1 (0.6 μM) was adsorbed on carbon-coated EM grids, washed and stained on a drop of 1% uranyl acetate solution. 

### 4.8. Gene Expression Analyses

For the screening of primary T1 transformants, total RNA was prepared from leaves of two week-old *L. japonicus* plants by the procedure reported by Kistner and Matamoros [76] and reversed-transcribed as described below. The oligonucleotides used for the PCR amplification are Aeq-for 5′-GCAAGCCAAACGACACAAAG-3′; Aeq-rev 5′-GAACCAACGCTCATCCGTAT-3′. The amplified 162 bp aequorin fragment was inserted in pCR2.1 cloning vector (Thermo Fisher Scientific) and confirmed by sequencing. The PCR program used was as follows: 94 °C for 5 min and 30 cycles of 94 °C for 30 s, 58 °C for 30 s, and 72 °C for 30 s. The ubiquitin gene (*UBI*, AW719589) was used as an internal standard to normalize expression levels (25 cycles of amplification; *UBI*-F-1 5′-TTCACCTTGTGCTCCGTCTTC-3′; *UBI*-R-1 5′-AACAACAGCACACACAGACAATCC-3′).Semi-quantitative RT-PCR analyses of defence gene expression in *L. japonicus* cell suspension cultures incubated in control conditions or after treatment with HYTLO1 were performed as recently described [75]. The defence-related genes encoding for the following proteins were chosen: MPK3, an enzyme frequently activated in signal transduction cascades in response to phytopathogens [77]; CP450, an enzyme involved in a wide range of biosynthetic reactions, including the synthesis of phytoalexins [78]; WRKY33, a transcriptional regulator involved in a large array of defence responses [79,80]; PR1, which expression is induced by many pathogens, and considered as a molecular marker of systemic acquired resistance (SAR) [81,82]. Transcript levels were normalized to *ATP synthase* [51]. The oligonucleotide primers were as described by [51] for *LjMPK3*, *LjWRKY33, LjCP450*, and *LjATPsyn*, and by [53] for *LjPR1*. The number of cycles exploited for a linear range of gene amplification in the RT-PCR reactions was experimentally determined. Densitometric analysis of agarose gels stained with 0.5 μg/mL ethidium bromide was carried out with the Quantity One software (Bio-Rad, Hercules, CA, USA). RT-PCR experiments were conducted in triplicate on at least three independent experiments. 

Quantitative reverse transcription PCR, used to validate the data, was performed in 10 μL using HOT FIREPol EvaGreen qPcr Mix Plus (Solys BioDyne, Tartu, Estonia) and 2.5 ng of the different cDNA template. Three replicates were performed for each reaction. The qPCR reaction was conducted in a 7500 Real-Time PCR System (Thermo Fisher) according to the following cycle: 95 °C for 12 min; 95 °C for 15 s, 59 °C for 20 s, for 40 cycles. Differences in the target genes expression were evaluated by a relative quantification method normalizing the data to the endogenous reference gene *LjATPsyn*.

### 4.9. Cell Viability

Cell viability, after treatment for HYTLO1 for different time intervals, in the absence or presence of Ca^2+^ chelating agents/Ca^2+^ channel inhibitors, was determined by the Evans Blue method [42]. 

### 4.10. Statistical Analysis

Data are expressed as mean ± SE. The statistical significance of differences (*P* < 0.05) between means was evaluated using the Student’s *t* test.

## Supplementary Materials

Supplementary materials can be found at http://www.mdpi.com/1422-0067/19/9/2596/s1.

## Abbreviations

Ca^2+^	Calcium
cADPR	Cyclic ADP-ribose
2,4-D	Dichlorophenoxyacetic acid
EGTA	Ethylene glycol-bis(β-aminoethyl ether)-*N*,*N*,*N*′,*N*′-tetraacetic acid
NAADP	Nicotinic acid adenine dinucleotide phosphate
TEM	Transmission electron microscopy
TPC	Two-pore channel
