# Supplementary material for: The Hydrophobin HYTLO1 Secreted by the Biocontrol Fungus Trichoderma longibrachiatum Triggers a NAADP-Mediated Calcium Signalling Pathway in Lotus japonicus
Source: Int J Mol Sci. 2018 Sep 1;19(9):2596. doi: 10.3390/ijms19092596 (PMC6164116; doi:10.3390/ijms19092596)
Supplement: Supplementary file 1 [file ijms-19-02596-s001.pdf]

A

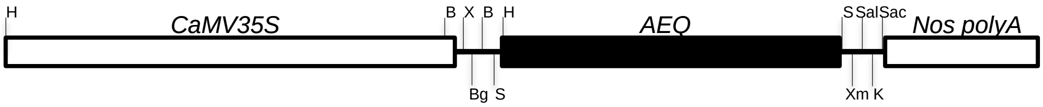

B

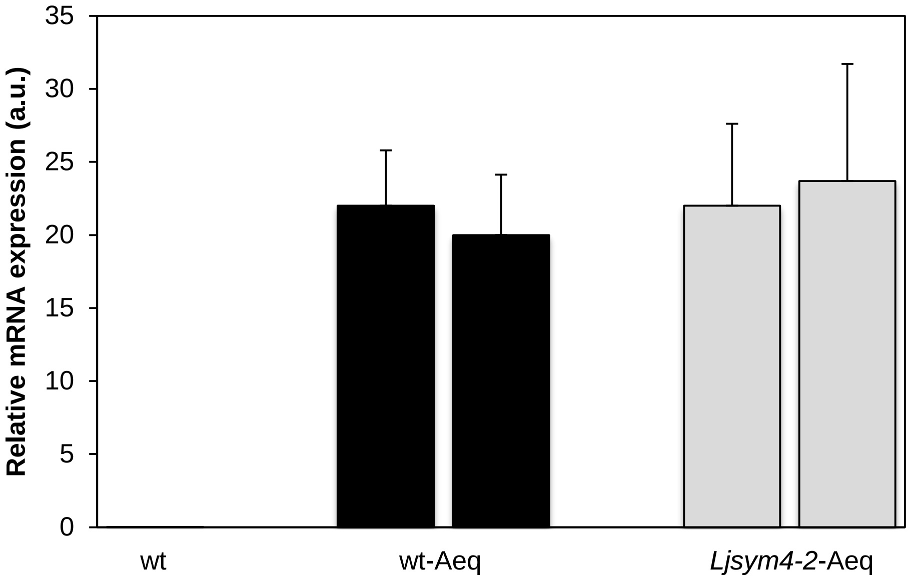

Figure S1. (**A**) Schematic map of the aequorin expressing cassette of pAB1 T-DNA construct. X, *Xba*I; Bg, *Bgl*II; B, *Bam*HI; S, *Sma*I; H, *Hind*III; Xm, *Xma*I; Sal, *Sal*I; K, *Kpn*I; Sac, *Sac*I. (**B**) Level of aequorin transcript in *L. japonicus* wild-type and *Ljsym4-2* mutant transformants of the T2 generation, selected to set up cell suspension cultures. Expression levels are normalized to that of the internal control ubiquitin. Data are the means ± SE of three independent experiments.

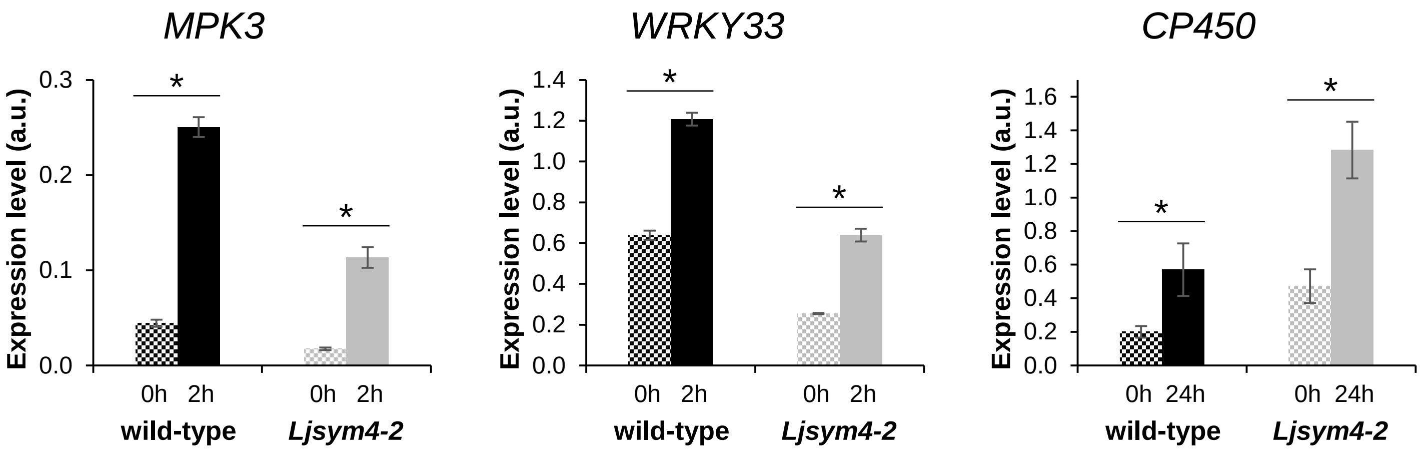

Figure S2. Quantitative RT-PCR analysis of *MPK3*, *WRKY33* and *CP450* transcripts in *L. japonicus* cell cultures of the wild-type line and *Ljsym4-2* mutant in control conditions (dotted bars) and after treatment with 0.6 µM HYTLO1 (solid bars) for different time intervals. Expression levels are normalized to the housekeeping gene *ATP synthase*. Data are the means ± SE of three replicates. * indicates statistically significant difference at *P*<0.05.

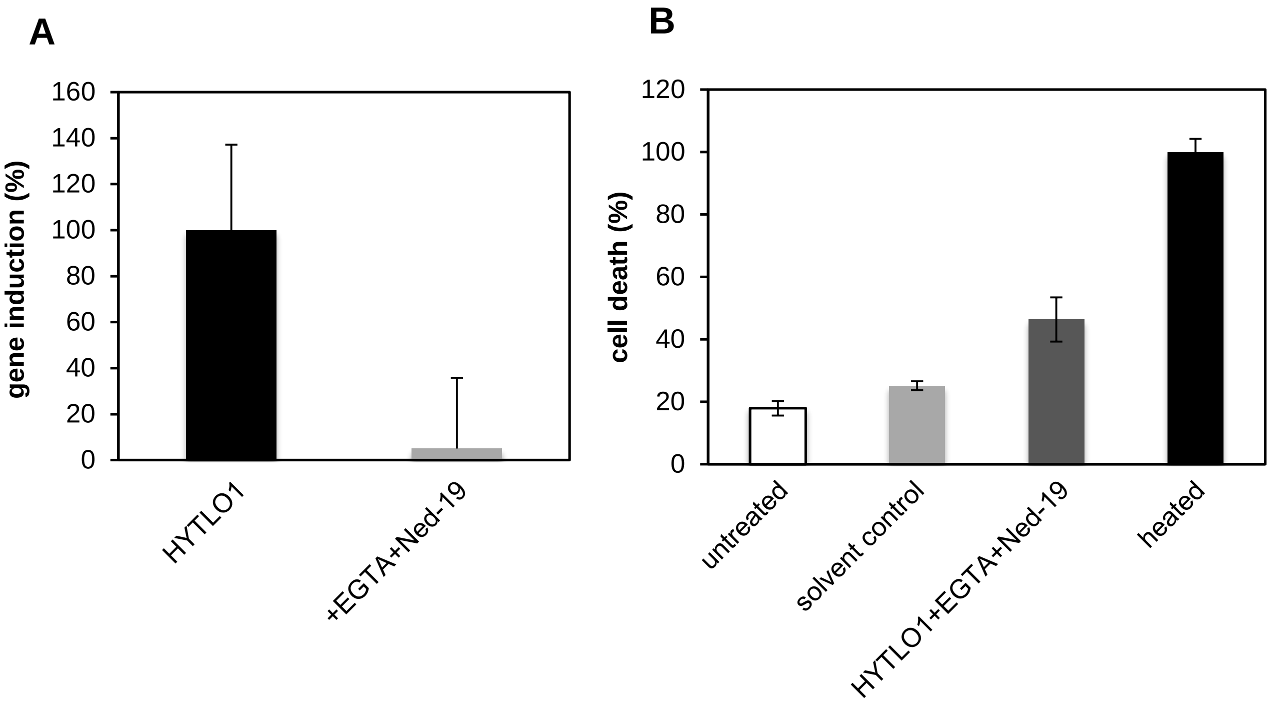

Figure S3. Effect of pretreatment with EGTA and Ned-19 on HYTLO1-induced *CP450* expression and cell viability in *L. japonicus* cells. (**A**) RT-PCR analysis of *CP450* expression after treatment with HYTLO1 (0.6 µM) for 24 h in regular cell culture medium (black bar) or after pre-treatment with 600 µM EGTA and 100 µM Ned-19 in Ca^{2+}-free medium (grey bar). (**B**) Cell viability assay after 24 h incubation in untreated samples (white bar), in Ca^{2+}-free medium supplemented with 600 µM EGTA and the solvents of Ned-19 (0.5% DMSO) and HYTLO1 (0.5% ethanol) (light grey bar), or in Ca^{2+}-free medium supplemented with 600 µM EGTA, 100 µM Ned-19 and 0.6 µM HYTLO1 (dark grey bar). 100% cell death corresponds to cells incubated for 20 min at 100°C (black bar). Data are the means ± SE of three independent experiments.

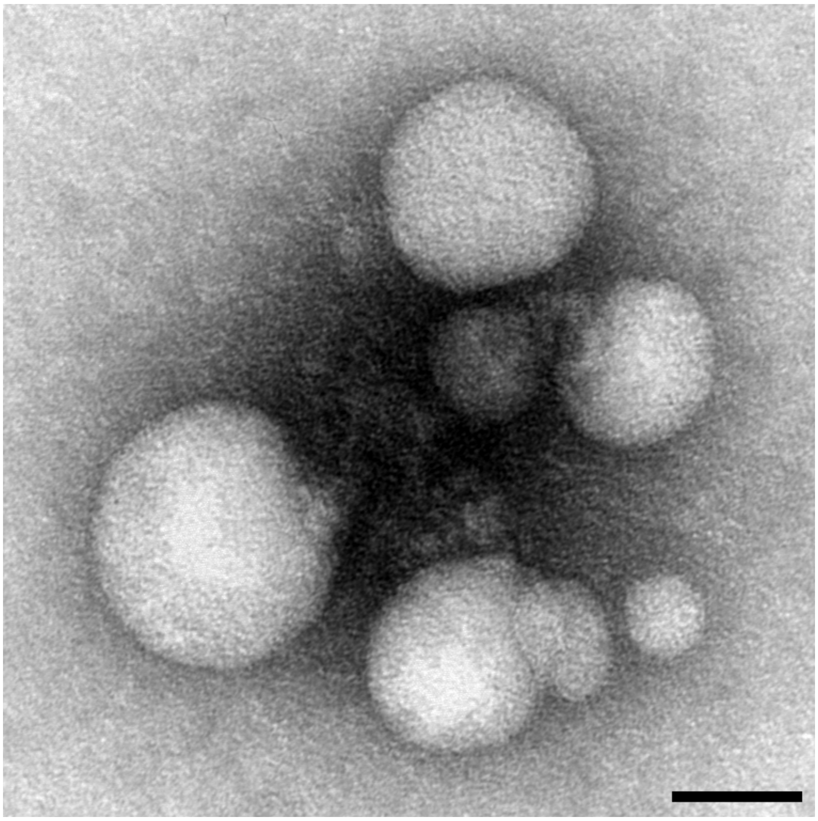

Figure S4. Negative staining with 1% uranyl acetate of purified HYTLO1. Bar, 50 nm.

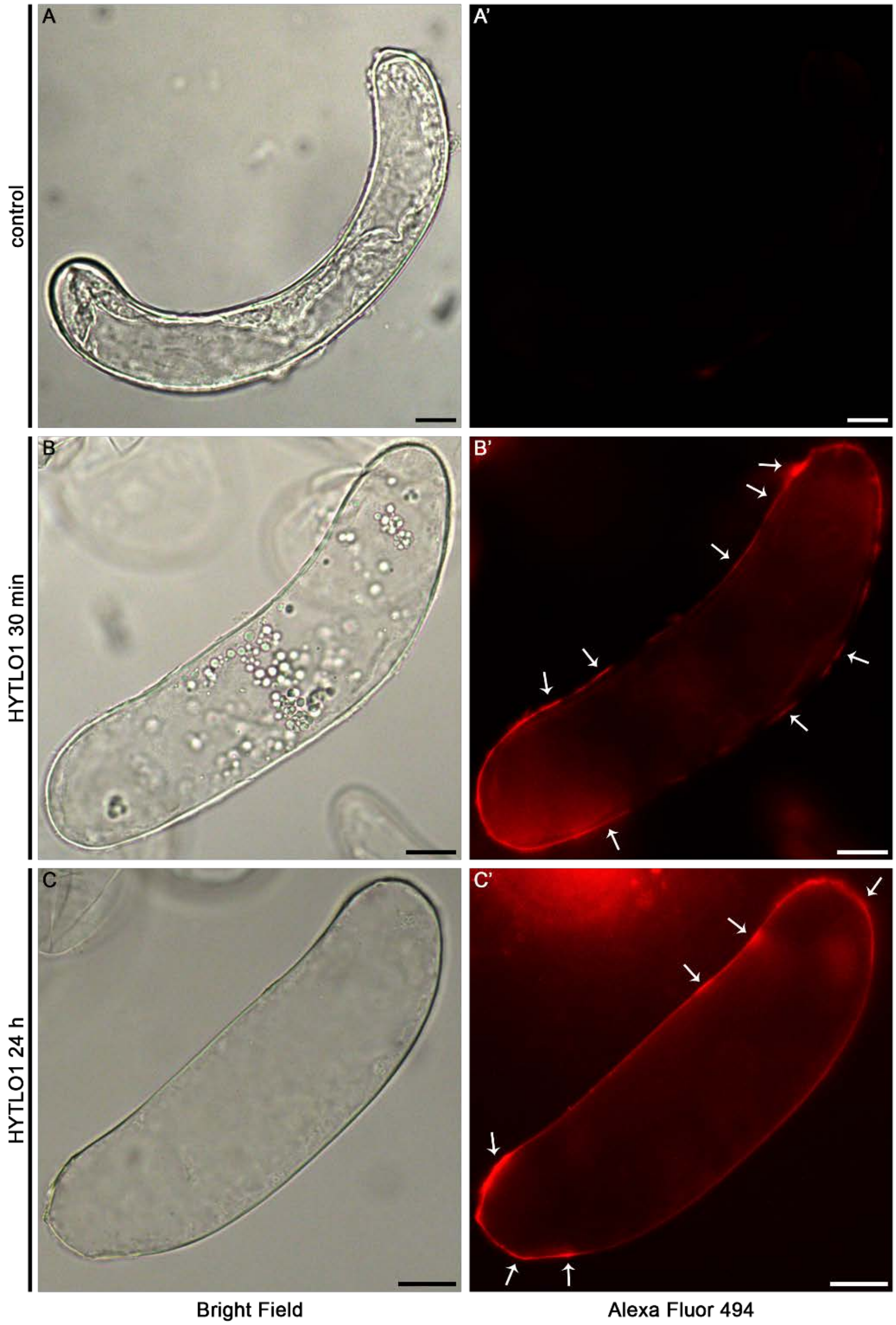

Figure S5. Immunofluorescence staining of *L. japonicus* cells treated with HYTLO1. Cells were incubated: (**A-A'**) in control conditions; (**B-B'**) with HYTLO1 (0.6 µM) for 30 min; (**C-C'**) with HYTLO1 (0.6 µM) for 24 h. After fixation, cells were incubated with an affinity-purified anti-HYTLO1 antibody followed by a red-fluorescent Alexa Fluor 494 secondary antibody. Bright field (**A-C**) and fluorescence microscopy (**A'-C'**) images of the same fields are shown. White arrows indicate an exclusive localization of the hydrophobin at the cell surface. Bars, 50 µm.

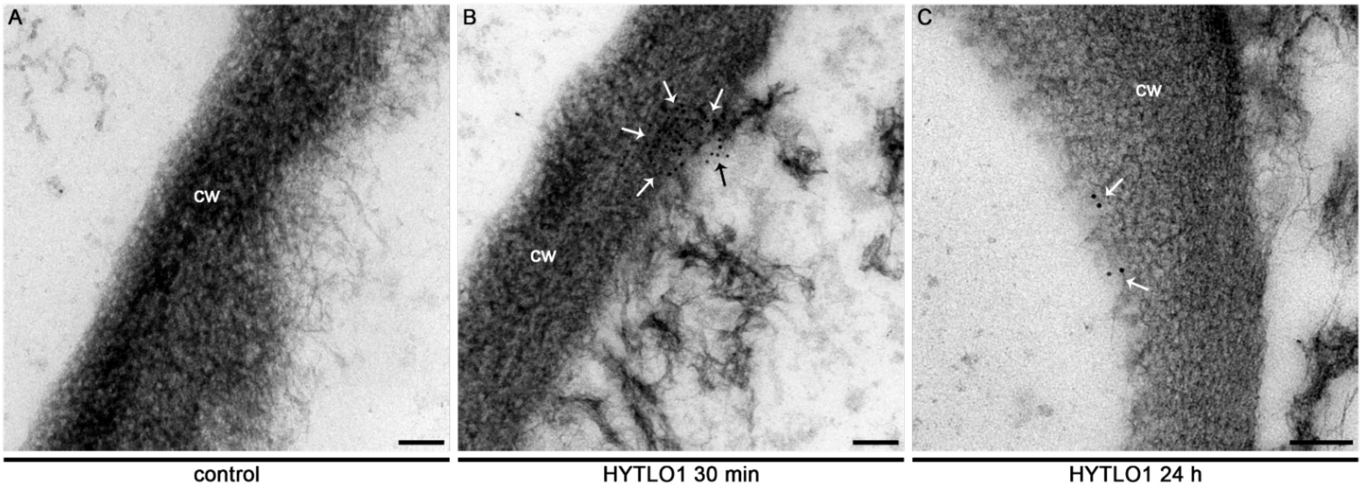

Figure S6. Immunocytochemical analysis of HYTLO1 in *Lotus japonicus* cells treated with the fungal hydrophobin. Untreated cells were used as negative control (**A**). Immunogold-labeled particles (arrows) are present across the plant cell wall width in 30 min-treated cells (**B**) and at the internal side of the cell wall in 24 h-treated cells (**C**). Bars, 100 nm.
